# Specific eradication of HIV-1 from infected cultured cells

**DOI:** 10.1186/1742-6405-7-31

**Published:** 2010-08-19

**Authors:** Aviad Levin, Zvi Hayouka, Assaf Friedler, Abraham Loyter

**Affiliations:** 1 Department of Biological Chemistry, The Alexander Silberman Institute of Life Sciences; The Hebrew University of Jerusalem, Safra Campus, Givat Ram, Jerusalem 91904, Israel; 2 Institute of Chemistry; The Hebrew University of Jerusalem, Safra Campus, Givat Ram, Jerusalem 91904, Israel

## Abstract

A correlation between increase in the integration of Human Immunodeficiency virus-1 (HIV-1) cDNA and cell death was previously established. Here we show that combination of peptides that stimulate integration together with the protease inhibitor Ro 31-8959 caused apoptotic cell death of HIV infected cells with total extermination of the virus. This combination did not have any effect on non-infected cells. Thus it appears that cell death is promoted only in the infected cells. It is our view that the results described in this work suggest a novel approach to specifically promote death of HIV-1 infected cells and thus may eventually be developed into a new and general anti-viral therapy.

## Findings

No cure or vaccine are currently available for the human immunodeficiency virus type 1 (HIV-1) infection as well as for the resulting Acquired Immunodeficiency syndrome (AIDS) [1]. However, an highly active antiretroviral therapy (HAART), which blocks the activities of the viral reverse transcriptase and protease and inhibits the virus-host fusion process, is presently used [2,3]. The HAART transforms the infection process into a chronic disease [4-6]. Furthermore, the risk of infection can significantly be reduced if the HAART treatment is given right after exposure to the virus [7].

New therapeutic approaches and new anti-viral inhibitors are being continuously developed to obtain a better restriction of the HIV-1 infection process [8-19]. However, once the viral cDNA is integrated into the host chromosome it is almost impossible to terminate infection process and cure AIDS. A way to eradicate the integrated viral cDNA from virus infected cells by stimulating the viral Integrase (IN) mediated disintegration process was suggested recently [20,21]. However, this approach is only in its initial steps [20].

HIV-1 infected cells, unlike cells infected by other retroviruses, bear only 1-2 copies of integrated viral cDNA/cell [22,23]. This is in spite of the presence of numerous copies of unintegrated viral cDNA [22,24]. Recently we have shown that this restriction is due to inhibition of the viral IN activity as well as of its nuclear import by an early expressed viral Rev protein following Rev-IN interaction [25-30]. Disruption of the Rev-IN complex by IN-derived cell permeable peptides, such as the INS [31] and INrs [28], results in multi-integration of the viral cDNA [26,28,31]. Previous findings have shown that multi-integration of viral DNA in AIDS patients may lead to host genome instability [32]. Indeed, a correlation between promotion of multi-integration and increase in cell death was demonstrated recently by us [25].

**Figure 1:**
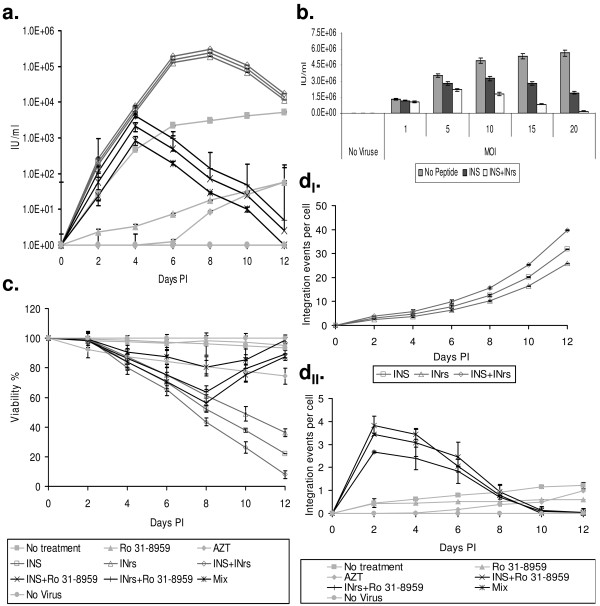
**Specific killing of HIV-1 infected cells**. **(a) **H9 lymphocyte T cells were infected by the WT HIV-1 at MOI of 0.1, exactly as described in [28], and then the infected cells were treated every two days with the indicated molecules or combinations. Every two days a sample was removed and its virus titer was estimated by the MAGI assay [37] using TZM-bl cells exactly as described [28]. **(b) **H9 lymphocyte T cells were infected with WT HIV-1 at the indicated MOIs and treated with INS or INS+INrs. The amount of virus production was estimated using MAGI assay on TZM-bl cells at 48 h PI. **(c) **Same as **(a) **but cells viability was estimated by the MTT assay as described in [28]. **(d**_**I**_**) **and **(d**_**II**_**) **Same as **(a) **but the average amount of viral cDNA integration events/cells was estimated by quantitative hemi-nested Real Time PCR exactly as described in [28]. Cells were grown as described in [28]. Viruses were produced and viral stock titer was estimated as described in [29]. Peptides were synthesized and purified as described in [28,31]. The following concentrations were used: AZT 2 μM, Ro 31-8959 10 nM, INS/INrs 150 μM. Every experiment was preformed at least three times with relative error not more ±10%. Error bars represent standard deviation.

Based on these observations we have developed a novel approach to specifically and significantly eradicate HIV-1 infected cells as well as to eliminate infectious virions from cultured cells. As can be seen in Fig. 1a, addition of the integration-stimulating INS or INr peptides or combination of both (150 μM each) to cells infected by wild type (WT) HIV-1 significantly increased the appearance of new virions during the first 6-8 days post infection (PI). However, from the eighth day PI, a decrease in virus production can be observed. The results in Fig. 1b show that the degree of the reduction is directly correlated to the MOI (multiplicity of infection) of the infected HIV-1. Almost complete eradication of virions was obtained when cells were infected, in the presence of the INS and the INr peptides, by relatively high titer of the virus (Fig. 1b). This eradication (Fig. 1a and 1b) is probably due to promotion of cell death (Fig. 1c), which in turn may result from the peptides induced stimulation of the integration process (Fig. 1d_I _and see [28]). Our results indicate that at very long periods PI a complete eradication of virus particles is obtained (Fig. 1).

When the specific HIV-1 protease inhibitor (Ro 31-8959 [33]) was added to virus infected cells together with either the INS or the INrs peptides or with both (the mixture of the INS, INrs and Ro 31-8959 was designated as Mix, see Fig. 1) the increase in virus production (Fig. 1a) and in viral cDNA integration (Fig. 1d_II_) was observed only during the first 2-4 days PI. On the other hand a drastic reduction in both virus production and cDNA integration could be observed from the fourth day PI and on, reaching below the detection levels in the presence of the Mix (Fig. 1a and 1d_II_). As can be seen (Fig. 1c) about 40% of the cultured cells died by the eighth day PI following the addition of the Mix. This percentage may represent the relative amount of virus infected cells, probably indicating total death of these cells. Furthermore, our results (Fig. 1a and 1d_II_) clearly show that at this time (8 days PI) the large majority of the virus was cleared from the culture. Therefore it is conceivable that the increase in the percentage of viable cells observed between 8-12 days PI (Fig. 1c) is due to division of uninfected cells.

To determine whether the above treatment (combination of INS + INrs and Ro 31-8959) indeed results in eradication of the infected virions and termination of the infection process, the following experiment was conducted: the cultured cells were infected by the WT HIV at MOI of 1 and 24 h PI cells were treated, every two days, with Ro 31-8959, INS+Ro 31-8959, INrs+Ro 31-8959 or by the Mix for the total duration of two weeks. Following this period the treated cells were left to grow, untreated, for two additional weeks.

**Figure 2:**
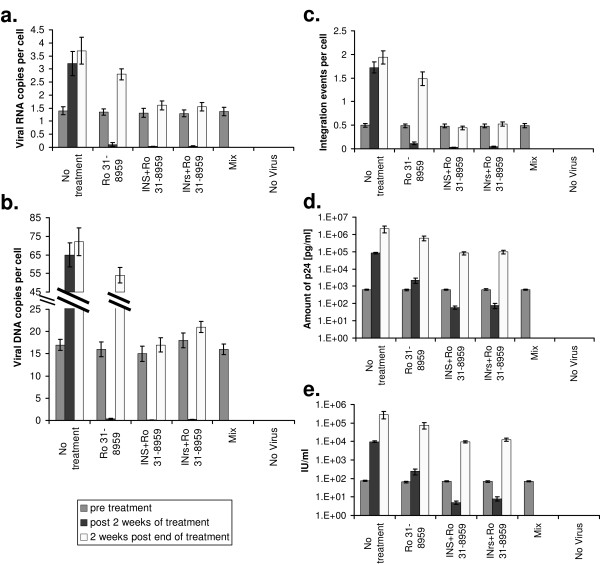
**Eradication of HIV-1 infection**. H9 lymphocyte T cells were infected with the WT HIV-1 at MOI of 1, exactly as described in [28]. Starting at 24 h PI the infected cells were treated every two days with the indicated molecules for two weeks. At the end of the two weeks treatment, cells were left to grow untreated. **(a) **The average amount of viral RNA copies per cell was estimated as described in [38]: prior to treatment, at the end of the two weeks treatments and two weeks post the termination of the treatment (four weeks PI). **(b) **Same as in **(a) **but the average amount of viral DNA copies per cell was estimated as described in [29,39]. **(c) **Same as in **(a) **but the average amount of integration events per cells was estimated as described in [28]. **(d) **Same as in **(a) **but the average amount of viral p24 was estimated as described in [30]**(e) **The amount of infectious virus produced by the cells was estimated, as described in [28]. All other conditions ad described in Fig. 1.

At the end of each of those periods namely: pre treatment, after two weeks of treatment and two weeks post termination of treatment (four weeks PI), the average amounts of viral RNA copies/cell (Fig. 2a), total viral DNA copies/cells (Fig. 2b) and of integrated viral cDNA/cell (Fig. 2c) as well as the amounts of viral p24 protein (Fig. 2d) and appearance of new virions (Fig. 2e) were estimated.

As can be seen a substantial reduction in virus production and integration was observed following the first two weeks treatment (Fig. 2), However, when treatment with the various combinations of peptides and the protease inhibitor was terminated, virus production and integration were restored except in cells treated with the Mix, indicating a Mix induced complete eradication of infection (Fig. 2).

**Figure 3:**
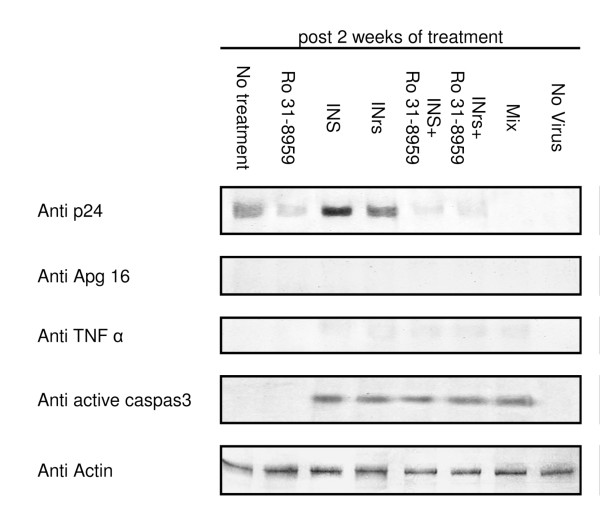
**Correlation between cell death and treatment**. H9 lymphocyte T cells were infected with the WT HIV-1 at MOI of 1, exactly as described in [28]. Starting at 24 h PI the infected cells were treated every two days with the indicated molecules for two weeks. Following the two weeks of treatment cells were lysed as described in [25,29] and the laysets were subjected to western blot analysis for actin as described in [25,29] for the cell death markers (Apg16 marker for autophagy [35], TNF α marker for necrosis [36] and active caspase 3 marker for apoptosis [34]) as described in [40] and for the viral p24 as described in [41].

It appears that the INS and INrs induced cells death is mostly by a caspase 3-dependent apoptosis pathway. This can be inferred from the western blot analysis which shows the appearance of active caspase 3 (apoptosis marker [34]) (Fig. 3). On the other hand, no autophagy cell death or necrosis could be observed following western blot analysis using the anti apg16 (autophagy marker [35]) or TNF α (necrosis marker [36]) respectively (Fig. 3). As expected--and also observed in Fig. 2d--a western blot analysis also show that there is reduction in the production of p24 in cells treated by the protease inhibitor Ro 31-8959 (Fig. 3). In addition both INS and INrs peptides which stimulate integration and infection also stimulated production of p24 (Fig. 3 and see also [28,31]). This increase in infection is in direct correlation to apoptotic cell death (Fig. 3 and see also [28,31]). On the other hand, when Ro 31-8959 was added together with INS, INrs or both, a significant decrease in p24 as well as increase in apoptotic cell death could be observed (Fig. 3).

It should be noted that the possibility in which a low number integrated viral DNA is still be present at a latent state cannot be totally excluded. Further experiments are presently being conducted in our laboratory in order to study reactivation of those few-- if any--latent proviruses.

We conclude that stimulation of viral integration by the INS and INrs peptides, combined with the prevention of virion production by the protease inhibitor, not only resulted in blocking of HIV-1 infection but also in extermination of the infected cells by invoking apoptosis. This treatment has cleared the cell culture from cells bearing the integrated proviruses. It should be added however that the novel approach described here for AIDS therapy is only in its initial steps and further attempts to improve the activity of the stimulating peptides are currently conducted in our laboratory.

## Competing interests

The authors declare that they have no competing interests.

## Authors' contributions

A. Levin designed and performed the experiments, analyzed data and contributed to writing the paper; ZH performed peptide synthesis and purification; AF designed the study, and contributed to the writing; A. Loyter designed the study, contributed to the writing of the paper and coordinated the study. All authors have read and approved the manuscript.
